# Exploring poly‐L‐lysine‐based particle capture for atomic force microscopy studies of extracellular vesicles

**DOI:** 10.1111/jmi.70060

**Published:** 2026-01-06

**Authors:** L. Conti, A. Ridolfi, A. Borup, M. J. C. van Herwijnen, P. Nejsum, M. H. M. Wauben, C. Albonetti, F. Valle, M. Brucale

**Affiliations:** ^1^ Consorzio Interuniversitario per lo Sviluppo dei Sistemi a Grande Interfase (CSGI) University of Florence Florence Italy; ^2^ Consiglio Nazionale delle Ricerche (CNR) Istituto per lo Studio dei Materiali Nanostrutturati (ISMN) University of Bologna Bologna Italy; ^3^ Department of Clinical Medicine Aarhus University Aarhus Denmark; ^4^ Department of Biomolecular Health Sciences Faculty of Veterinary Medicine Utrecht University Utrecht The Netherlands

**Keywords:** atomic force microscopy, extracellular vesicles, morphometry, nanomechanics

## Abstract

We herein investigate the effects of varying the main experimental variables in one of the most used protocols for extracellular vesicle (EV) immobilisation on substrates for subsequent atomic force microscopy (AFM) quantitative morphometry and nanoindentation performed in liquid. We introduce the parameter *Q* as a quantitative measure of total adsorbed material and show how it can be used as an estimator of relative sample concentrations across multiple AFM imaging experiments. We show how *Q* is logarithmically dependent on substrate charge density, whereas the EV contact angle (CA) surprisingly does not follow the same dependence. Finally, we propose an optimised protocol for AFM quantitative morphometry in air that yields the same EV size distributions obtained in liquid.

## LAY DESCRIPTION

Extracellular vesicles (EVs). Over the past 10 years, interest in EVs has grown significantly, establishing this research field as an extremely fertile area of investigation across multiple fields. Still, EV isolation and characterisation pose nontrivial technical challenges, some of which can be addressed via single‐particle characterisation techniques.

Atomic force microscopy (AFM) is a widely adopted tool for measuring EV morphology and nanomechanics at the single‐particle level. These measurements require EVs to be immobilised on a surface; given that natural EVs possess a negative surface charge, a common strategy involves depositing them on substrates functionalised with poly‐L‐lysine (PLL), a positively charged polymer.

Although widely adopted, the specific mechanisms and effectiveness of PLL‐based EV capture have not been systematically examined in earlier studies. In this paper, we explore how the main variables of PLL substrate functionalisation impact EV adsorption and, consequently, the outcomes of AFM imaging. We show how the geometry of surface‐bound vesicles is not affected by the amount of PLL employed for substrate functionalisation. We then present an optimised protocol for EV immobilisation, along with its underlying rationale. Utilising this protocol, it is possible to estimate the relative concentrations of EV samples via their particle surface density as measured by AFM. Finally, we demonstrate that EV size distributions obtained through AFM imaging in liquid can be reliably reproduced in air, provided a few straightforward precautions are taken.

## 1 INTRODUCTION

Extracellular vesicles (EVs) are biogenic, ubiquitous membrane‐bound particles playing essential roles in numerous intercellular communication networks. Over the past 10 years, interest in EVs has grown significantly,^1^, ^2^ establishing this research field as an extremely fertile area of investigation across multiple fields.^3^, ^4^, ^5^, ^6^ However, EV studies still involve nontrivial technical challenges, as demonstrated by the periodic updating and refinement of the Minimal information for studies of extracellular vesicles (MISEV) guidelines,^7^, ^8^, ^9^ which represent a snapshot of currently available techniques for EV production, enrichment and characterisation as well as of their limitations. Most of these technical challenges are the direct result of the EVs’ complex physico‐chemical features,^10^ which include compositional and dimensional heterogeneity, environmental susceptibility/adaptivity, and complex structural dynamics. In this context, single‐particle characterisation techniques^11^, ^12^, ^13^, ^14^ seem to be especially promising in attempting to investigate the intrinsic complexity of EV populations. Scanning and transmission electron microscopies (EM) in general, and cryogenic‐EM in particular, proved especially useful for elucidating EV size and ultrastructure.^15^, ^16^, ^17^ A widespread cheaper alternative is represented by atomic force microscopy (AFM), which combines specific limitations with unique advantages for the study of EVs.^18^, ^19^, ^20^

The main technical advantages of AFM in the context of EV research are that samples can be observed in quasi‐physiological conditions without labelling, and that it is possible to infer nanomechanical information about individual particles.^19^, ^21^, ^22^ Moreover, the accuracy and resolution of current entry‐level commercially available AFM are sufficient to perform single‐particle quantitative morphometry^23^ on all but the smallest (e.g. HDLs) particles typically found in EV isolates,^24^, ^25^ and in some cases to detect ultrastructural details of individual EVs.^26^ Quantitative morphometry simultaneously determines single‐particle size distributions closely resembling those obtained via cryo‐EM^24^ and gives an estimate of the mechanical stiffness of individual vesicles, thus enabling the distinction between EVs and co‐isolates in complex mixed samples.^24^, ^27^, ^28^

AFM can also be used as a nanoindenter^29^ to quantify the mechanical response of individual particles to an applied stress.^19^, ^20^ Intact vesicles display a linear indentation regime, whose slope corresponds to the vesicle's stiffness, which distinguishes them from the majority of non‐vesicular particles, as well as a peculiar tether elongation plateau that correlates with internal pressurisation. Stiffness and tether force can be leveraged to calculate the bending moduli of individual vesicles via a model based on Canham–Helfrich theory.^30^ Interestingly however, there is still no consensus on which mechanical model should be employed to accurately describe the behaviour of EVs.^31^, ^32^

Despite the availability of techniques such as those mentioned above, simple imaging remains the most widespread AFM technique found in EV literature, probably for its combination of speed and convenience. However, AFM micrographs of EVs need to comply to specific requirements to be useful for quantitative morphometry; the most important arguably being the method employed to deposit the sample on an appropriate substrate. One of the most widespread methods for anchoring EVs to a surface is to leverage their negative surface potential by depositing the positively charged polymer poly‐L‐lysine (PLL) on a substrate, closely following widely employed cell immobilisation protocols.

Herein, we will investigate how the main variables of PLL substrate functionalisation influence the adsorption of EVs and, ultimately, the results of AFM quantitative morphometry. We describe an optimised protocol for EV immobilisation and its rationale; lastly, we show how EV size distributions obtained via AFM imaging in liquid can be replicated via imaging in air, as long as a few simple precautions are observed.

## 2 MATERIALS AND METHODS

### 2.1 Extracellular vesicle enrichment

Since this study focuses on the colloidal, morphological and mechanical characteristics of EVs exclusively, we chose to only employ samples that were previously characterised extensively by our groups. We used EVs enriched from the parasitic nematode *Ascaris suum* excretory/secretory products following the ultracentrifuge‐based (‘UC’) protocol described in a previous study,^33^ and EVs enriched as described elsewhere^23^ from raw bovine milk. For both samples, total particle count as estimated via Nanoparticle Tracking Analysis was ∼10^11^ particles/mL.^26^, ^33^, ^34^

### 2.2 Poly‐L‐lysine substrate preparation

Poly‐L‐lysine (PLL)‐coated glass coverslips were prepared as follows. Microscopy glass slides (15 mm diameter round coverslips, thickness ∼0.15 mm, Menzel Gläser) were first immersed for 2 h in a 3:1 (v:v) 96% H_2_SO_4_/30% H_2_O_2_ ‘piranha’ solution, then rinsed extensively in ultrapure water, cleaned in a sonicator bath (Elmasonic Elma S30H) for 30 min in acetone, followed by 30 min in isopropanol and 30 min in ultrapure water, and finally activated with air plasma for 5 min followed by immediate immersion in ultrapure water. Several different air plasma systems were tested, including, for example, Pelco EasiGlow (0.3 mbar air pressure, 30 mA current, 5 min plasma time) and Gambetti Kenologia Srl Colibrì (0.5 mbar air pressure, 300 s plasma time, 100 W power setpoint) leading to the same results, suggesting that the choice of specific plasma cleaner conditions is not critical.

Clean coverslips were then incubated for 30 min at room temperature in 0.5 mL of a freshly prepared solution of PLL (MW 70–150 kDa) dissolved in 100 mM, pH 8.5 borate buffer at room temperature; PLL concentration varied between 0.2 and 0.0001 mg/mL. After PLL incubation, glass slides were thoroughly rinsed with ultrapure water and dried with a gentle nitrogen flow, then used immediately for AFM studies. All reagents were acquired from Sigma‐Aldrich Inc (www.sigmaaldrich.com) unless otherwise stated.

### 2.3 EV sample deposition for AFM imaging in liquid

For all AFM experiments performed in liquid, 5 µL of the chosen EV preparation were mixed with 20 µL of microfiltered (pore size 0.2 µm regenerated cellulose syringe filters, Corning) PBS, deposited on a freshly prepared PLL‐functionalised glass slide and left to adsorb for 30 min at 4°C, then inserted in the AFM fluid cell (see below) without further rinsing. The concentration of each vesicle‐containing solution was adjusted by trial and error in successive depositions in order to maximise the surface density of isolated, individual vesicles and minimise clusters of adjoining vesicles. In one of the experiment series, successive depositions were made at increasingly high dilutions by adding more PBS, resulting in concentrations of 0.2×, 0.1×, 0.05× and 0.02× times the undiluted stock sample.

### 2.4 EV sample deposition for AFM imaging in air

For AFM imaging in air on mica, 2 µL of the stock sample were deposited on freshly cleaved ruby red mica and left to evaporate at RT for 1 h, then brought to complete dryness via a gentle nitrogen flow.

AFM imaging in air was also performed on PLL‐functionalised glass slides prepared exactly as described in the previous paragraph; however, instead of being inserted into the fluid cell without rinsing, samples were treated as follows before imaging. 200 µL of ultrapure water was added to the sample droplet resting on the substrate, left to equilibrate for 1 min, then quickly removed from the substrate by turning it upside down. Substrates were then fully dried via a gentle nitrogen flow. In some cases, the rinsing step was repeated multiple times before imaging.

### 2.5 AFM imaging, nanoindentation, and data analysis

Most atomic force microscopy (AFM) experiments were conducted in liquid at room temperature using a Bruker Multimode 8 system equipped with Nanoscope V electronics, a sealed fluid cell, and a JV piezoelectric scanner. The experiments utilised Bruker SNL‐A probes (triangular cantilever, nominal tip curvature radius of 2–12 nm, nominal elastic constant of 0.35 N/m), which were calibrated using the thermal noise method.^35^ A minority of the AFM experiments were performed in air, the only difference being that an air probe holder was used instead of a sealed fluid cell.

**FIGURE 1:**
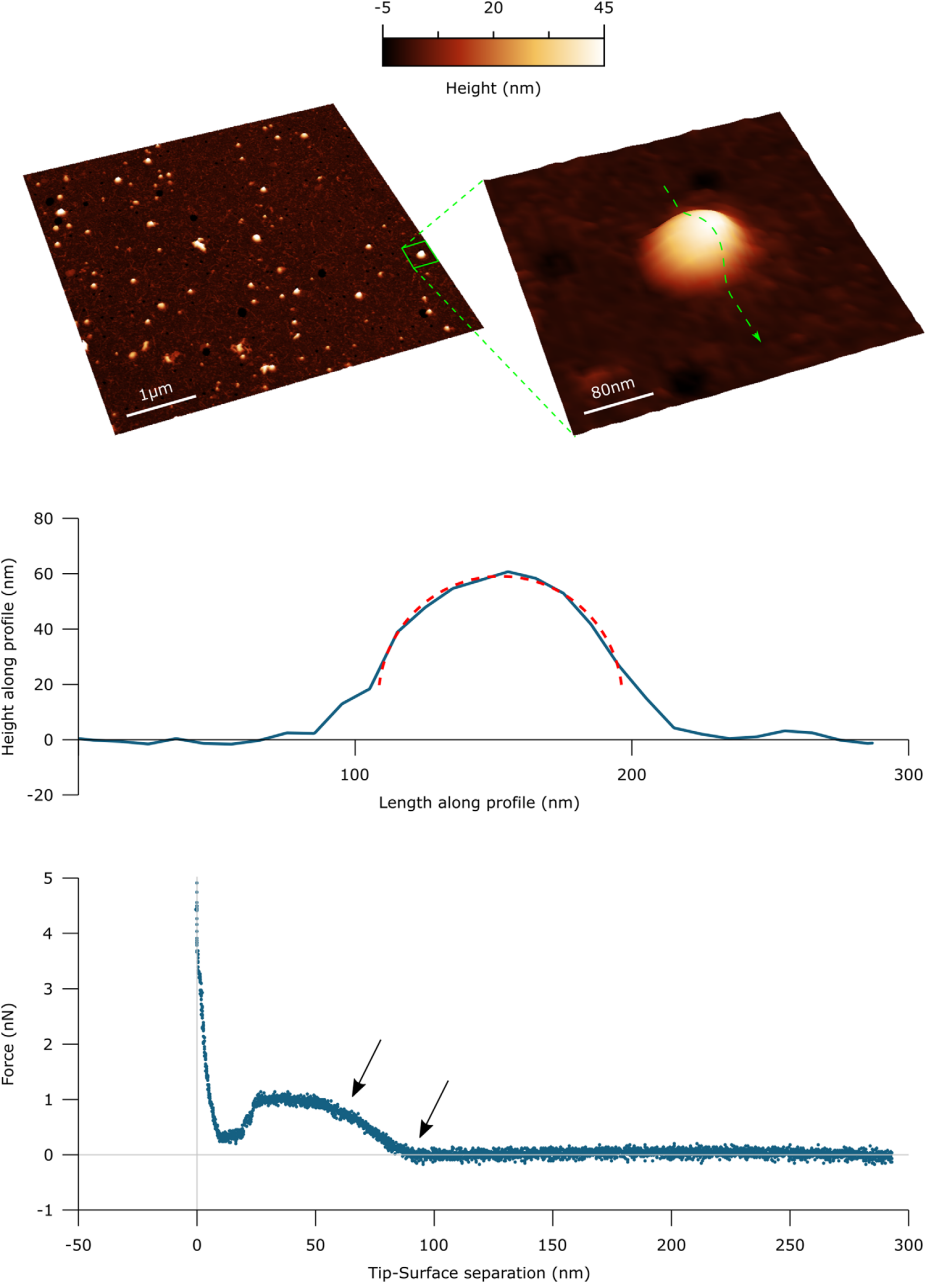
Top left: Example 5×5 µm AFM micrograph of *Ascaris suum* EVs deposited on a PLL‐functionalised glass substrate. (Top right): 400 × 400 nm magnification of an individual adsorbed EV, showing the typical spherical cap morphology. Middle: Example EV height profile as measured along the green dashed path in the top right panel. The red dashed line is a circular arc fit of the profile excluding points within 20 nm of the surface. Bottom: Typical nanoindentation force response of an individual EV. Intact vesicles show a characteristic linear regime immediately after the contact point (portion between the two black arrows). The slope of this portion of the curve corresponds to the vesicle's stiffness.

Imaging was carried out in PeakForce mode. To minimise vesicle deformation or rupture upon interaction with the probe, the applied force setpoint was kept below 400 pN and the lateral probe velocity was not allowed to exceed 5 µm/s. The feedback gain was set higher than the values typically used for optimal image quality to ensure minimal probe‐induced vesicle deformation during lateral contact along the fast scan axis. This resulted in images with comparatively high noise levels in the empty areas of the surface (≤20 nm peak to peak), but in which the height profiles of individual vesicles measured along both the slow and the fast scan axis could be fitted extremely well with circular arcs (see e.g. Figure 1). The average height value of all bare substrate zones was taken as the baseline zero height reference.

Quantitative morphometry was performed with a combination of Gwyddion 2.61^36^ and custom Python scripts to recover the surface contact angle (CA) and equivalent solution diameter (*sD*) of individual particles^23^ as well as their surface area and volume.

AFM nanoindentation was performed as described elsewhere.^23^

## 3 RESULTS AND DISCUSSION

PLL is an L‐lysine homopolypeptide that is widely used to immobilise cells on glass and plastic surfaces via electrostatic interactions. Its net positive charge per residue remains close to ∼1 for pH values below 9, undergoes a rapid sigmoidal transition centred at pH 10.6, and is close to 0 for pH values above 12.^37^ Since the vast majority of EVs isolated from natural sources have a negative surface charge,^38^, ^39^, ^40^ they can adhere to PLL‐functionalised surfaces at near‐physiological pH values by means of electrostatic interactions exerting an attractive force between their membrane and PLL.

Prior to that, PLL needs to interact with negative charges on the substrate for the functionalisation to be successful. The cleaning procedure we use (see Section 2) involves the complete oxidation of glass surface moieties during the air plasma cleaning step; subsequent immediate immersion in ultrapure water maximises the surface density of hydroxyl groups on the glass substrate and thus PLL adsorption.

In this study, we employed PLL with a declared MW range of 70–150 kDa, corresponding to around 500–1000 lysine residues and a contour length of 175–350 nm. This means that the amounts of PLL used for substrate functionalisation (0.5 mL of PLL solutions between 0.0001 and 0.2 mg/mL PLL, see Section 2.2) correspond to a range of approximately 30–600 µg of PLL per cm^2^ of glass, corresponding to between 2 × 10^16^ and 4 × 10^17^ monomeric lysine moieties per cm^2^ and thus between 10^2^ and 10^3^ positive charges per square nanometer. Since obtaining these surface densities is implausible, it seems reasonable to assume that only a small fraction of the lysine moieties actually interacts with available adhesion points present on the substrate during functionalisation.

At all the employed deposition concentrations (0.2–0.001 mg/mL), the effect of PLL functionalisation alone caused no discernible morphological changes with respect to bare glass surfaces. When observed in liquid, functionalised substrates were completely devoid of observable features and retained surface area roughness values of *Sq* ≈ 1.5 nm, coincident to those of non‐functionalised glass substrates (data not shown).

The deposition of EV isolates on PLL‐functionalised substrates typically shows a homogeneous distribution of particles on the surface (Figure 1). A large proportion of these particles shows curved profiles closely fitted by circular arcs (typical *R* ^2^ ≥ 0.95) above a certain distance from the substrate, where amorphous material accretion becomes irrelevant. When subjected to an applied force via nanoindentation, the vast majority of these domed particles show a linear contact mechanics regime that is typical of intact vesicles.^19^, ^20^, ^21^, ^22^, ^23^, ^30^, ^31^, ^41^, ^42^

As described elsewhere,^23^, ^24^, ^43^ AFM micrographs can be used for quantitative AFM morphometry, thus obtaining size and stiffness distributions of deposited particles. It is also possible to measure the total volume of adsorbed particles per square micron (*Q*) in a given AFM image.^24^, ^27^, ^28^, ^33^ This value cannot be reliably linked to specific absolute concentrations of the deposited samples; nonetheless, it is still quantitatively linked to the amount of adsorbed particles and thus to the relative concentrations of deposited samples.

**FIGURE 2:**
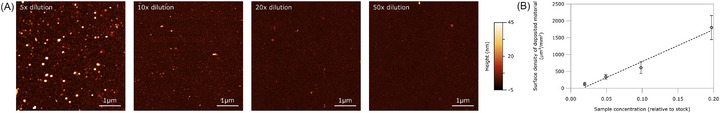
(A) Representative 5×5 µm AFM micrographs of aliquots from a single *Ascaris suum* EV preparation deposited at various sample dilutions on identical PLL‐functionalised substrates. As sample dilution increases, the resulting surface density of deposited particles decreases. (B) Average surface density of deposited particles as measured from multiple AFM micrographs taken at each successive dilution of the stock sample in PBS, as exemplified in panel A. Deposited sample concentration is expressed as a fraction of the starting stock sample (e.g. a fivefold dilution is reported as concentration = 0.2); all samples were deposited on glass slides functionalised with the same PLL concentration (0.01 mg/mL). Surface densities are expressed as the total volume of deposited particles per 1×1 mm^2^ area. Error bars represent the standard deviation of surface densities measured on five 5 × 5 µm AFM micrographs. The amount of deposited material is linearly dependent on sample concentration (dashed line: linear fit, *R* ^2^ = 0.97).

In order to ascertain the effect of varying EV sample concentrations on the deposition procedure, we performed a series of experiments at varying sample dilutions, calculating the average amount of deposited particles *Q* on a set of five 5 × 5 µm2 AFM images for each dilution (Figure 2A). For all the depositions, glass slides were functionalised with PLL at a concentration of 0.01 mg/mL. The deposited EV samples in this series were aliquots taken from the same *A. suum* batch, diluted in PBS between 5 and 50 times with respect to the stock, and incubated on substrates for 30 min. The calculated values of *Q* at varying sample concentrations (Figure 2B) exhibit a linear trend (*R* ^2^ = 0.97).

We previously reported on a similar experiment performed on varying amounts of ultracentrifuged plasma,^24^ also evidencing a linear correlation between sample concentration and particle surface density. Taken together, these experiments suggest that if all the AFM images show well resolved, non‐adjoining particles, and if *Q* follows a linear trend at all the explored concentrations, it is reasonable to assume that the surface was not saturated by sample particles, and their relative surface densities can thus be used to calculate the relative concentrations of deposited samples. In our experience, extending deposition times beyond 30 min do not lead to an increased amount of adsorbed particles, suggesting that in our deposition conditions, the vast majority of vesicles and co‐isolates adsorbs on the surface within this timeframe.

**FIGURE 3:**
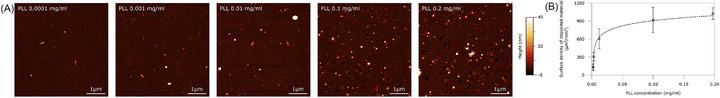
(A) Representative 5×5 µm AFM micrographs of aliquots from a single *Ascaris suum* EV preparation deposited on glass substrates functionalised with varying amounts of PLL. The resulting surface density of deposited particles is dependent on PLL concentration. (B) Average surface density of deposited particles as measured from multiple AFM micrographs of samples obtained by depositing aliquots from a single *Ascaris suum* EV sample on glass substrates functionalised with different PLL concentrations, as exemplified in panel A. Surface densities are expressed as the total volume of deposited particles per 1 × 1 mm^2^ area. Error bars represent the standard deviation of surface densities measured on five 5 × 5 µm AFM micrographs. The amount of deposited material is logarithmically dependent on sample concentration (dashed line: logarithmic fit, *R* ^2^ = 0.99).

We then performed a similar series of experiments, this time keeping constant the EV sample dilution (at 1:10 in microfiltered PBS) but varying the concentration of PLL during substrate functionalisation. Representative images of *A. suum* EVs deposited on substrates functionalised with PLL 1 × 10^−4^, 1 × 10^−3^, 1 × 10^−2^, 1 × 10^−1^ and 2 × 10^−1^ mg/mL show putative EVs with increasing surface densities (Figure 3A), apparently without reaching surface saturation even at the highest PLL concentration. However, in this case the correlation between PLL concentration and *Q* (Figure 3B) is best fitted by a logarithmic trend (*R* ^2^ = 0.99). From a merely practical perspective, PLL 0.01 mg/mL seems to represent the best compromise between maximum *Q* and low PLL usage.

Even though we cannot directly detect PLL on the substrate, knowing that *Q* is linearly correlated to sample concentration strongly suggests that the observed nonlinear dependence (Figure 3B) originates from the amount of adsorbed PLL at various functionalisation concentrations. While the logarithmic trend is compatible with a classic Langmuir isotherm model in which lysine charged residues compete for a limited number of anchoring points on the substrate, alternative explanations might also be possible. For example, at low PLL concentrations, each polymer chain might be free to interact with the substrate, leading to a compact layer of PLL molecules whose charges are more shielded by those of opposite sign present on the substrate. Conversely, at high PLL concentrations, individual polymer molecules might on average attach to the surface with a smaller number of residues per chain, leaving a higher proportion of charged residues available for particle capture. More experiments will be needed to elucidate the exact mechanism of EV/PLL interaction in this context; however, one immediate consequence of these results is that they prove it is important to perform any experiment used to compare *Q* values across multiple depositions on substrates functionalised with the same amount of PLL.

To evaluate the nanomechanical characteristics of individual deposited particles via AFM morphometry, we use contact angle (CA) as a quantitative geometrical shape descriptor, as described elsewhere^23^, ^24^ and applied to EV samples from several different natural sources.^26^, ^27^, ^28^, ^44^ Upon interaction with the PLL‐functionalised substrate, attractive forces are established between individual vesicles and the surface. These forces deform the spherical average shape of unadsorbed EVs into prolate shapes that can be closely approximated by spherical caps. This deformation is countered by each vesicle's stiffness; the stiffer a vesicle, the smaller the deformation it will undergo on a given substrate. The amount of deformation a vesicle undergoes can be quantitatively described via its CA, which can be experimentally determined via AFM morphometry. The average CA of a homogeneous population of vesicles is thus linked to their average stiffness, as verifiable via tandem nanoindentation/morphometry experiments.^23^ Particles plotted on a graph according to their spherical diameter (*sD*) and CA typically tend to cluster in different zones according to their biological identity.^24^, ^44^

Since average CA values are the result of two mutually opposing factors (stiffness and adhesion force), and since it seems reasonable to assume that the prevailing adhesion forces acting on vesicles deposited on PLL are electrostatic, a legitimate concern arises regarding CA reproducibility and comparability across different depositions. Due to this, we always made sure to only compare CA values of EV samples deposited following the exact same substrate functionalisation protocol,^24^ in an attempt to minimise PLL surface density variability.

**FIGURE 4:**
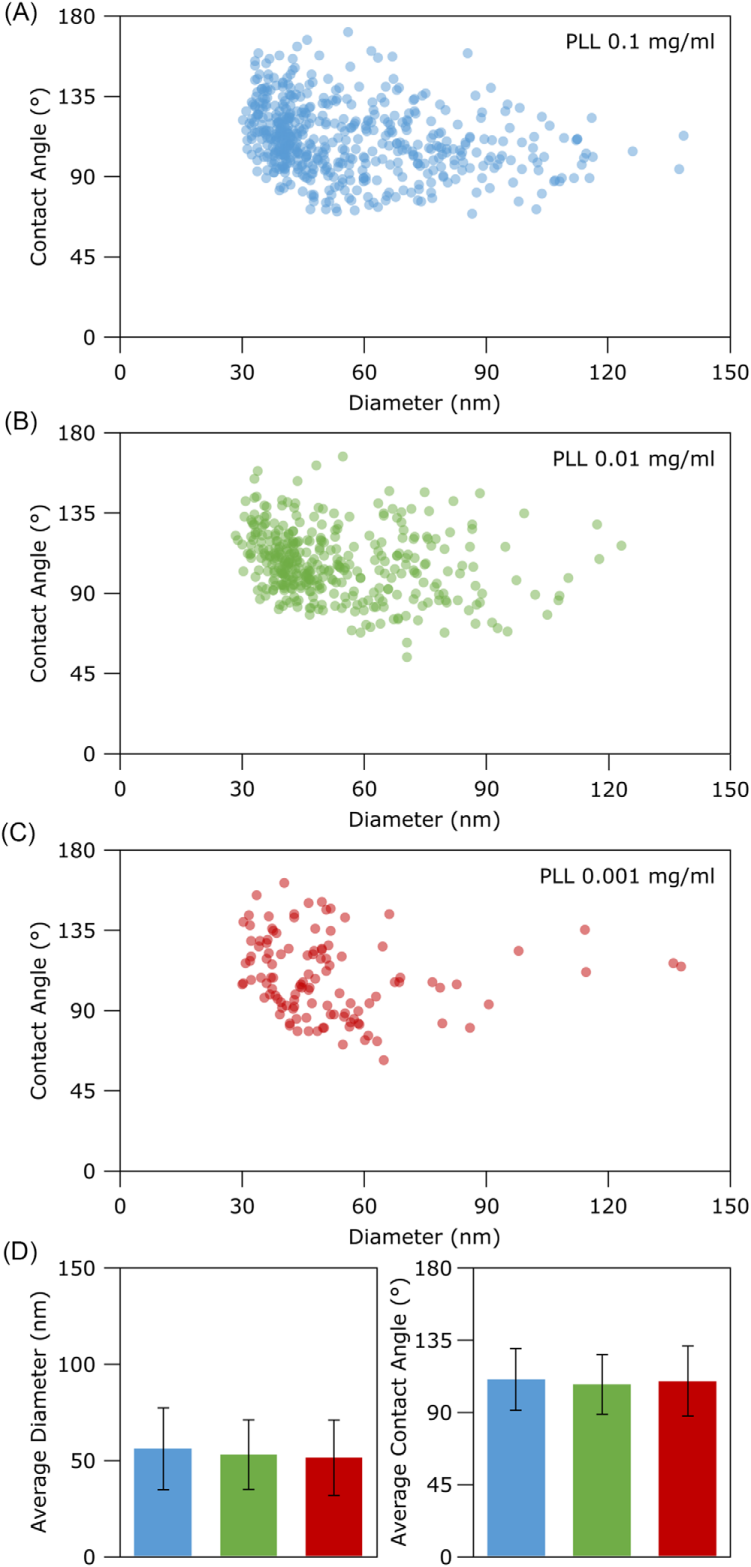
(A–C) Single‐particle quantitative morphometry of individual particles found in aliquots from the same *Ascaris suum* EVs preparation deposited on glass substrates functionalised with PLL at various concentrations. Each dot represents one particle. Contact angle and equivalent spherical diameter at constant surface were calculated as described elsewhere.^23^ (D) Average diameter (left) and contact angle (right) as calculated from scatterplot A (blue), B (green) and C (red). Despite the fact that varying PLL concentrations result in different amounts of adsorbed material, the morphometry of individual particles appears to be largely unaffected.

To quantify the magnitude of CA variation induced by functionalisation at different PLL concentrations, we performed quantitative morphometry on particles adsorbed on substrates prepared with 1 × 10^−3^, 1 × 10^−2^, 1 × 10^−1 ^mg/mL PLL (Figure 4). Surprisingly, the average CA values of adsorbed particles were coincident in all three samples (PLL 1 × 10^−3^ mg/mL: CA = 110 ± 19°, PLL 1 × 10^−2^ mg/mL: CA = 107 ± 18°, PLL 1 × 10^−1 ^mg/mL: CA = 109±22°). Average particle sizes also remained constant (see Figure 4). The same PLL concentrations were however responsible for the adsorption of markedly different particle amounts (Figure 3B), with *Q* at PLL 1 × 10^−1 ^mg/mL being ∼300% of that at PLL 1 × 10^−3 ^mg/mL.

This result is still challenging to rationalise given the currently available information; a tentative explanation might be that while the extent of substrate/sample electrostatic interactions are extremely important for particle capture during deposition (thus affecting *Q*), adhesion forces acting upon vesicles resting on the surface are then dominated by other factors such as van der Waals forces.^45^

**FIGURE 5:**
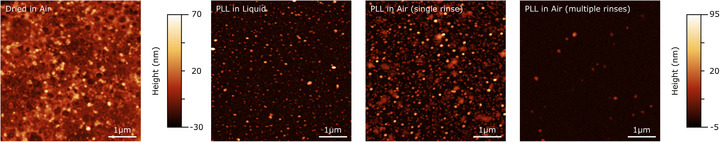
Representative 5 × 5 µm AFM micrographs of aliquots from a single bovine milk EV preparation. From left to right: air imaging of a 2 µL aliquot of the stock sample dried on freshly cleaved mica; liquid imaging on 0.01 mg/mL PLL‐functionalised substrate; air imaging on a duplicate sample with single rinse; air imaging on a duplicate sample subjected to multiple rinses with ultrapure water.

**FIGURE 6:**
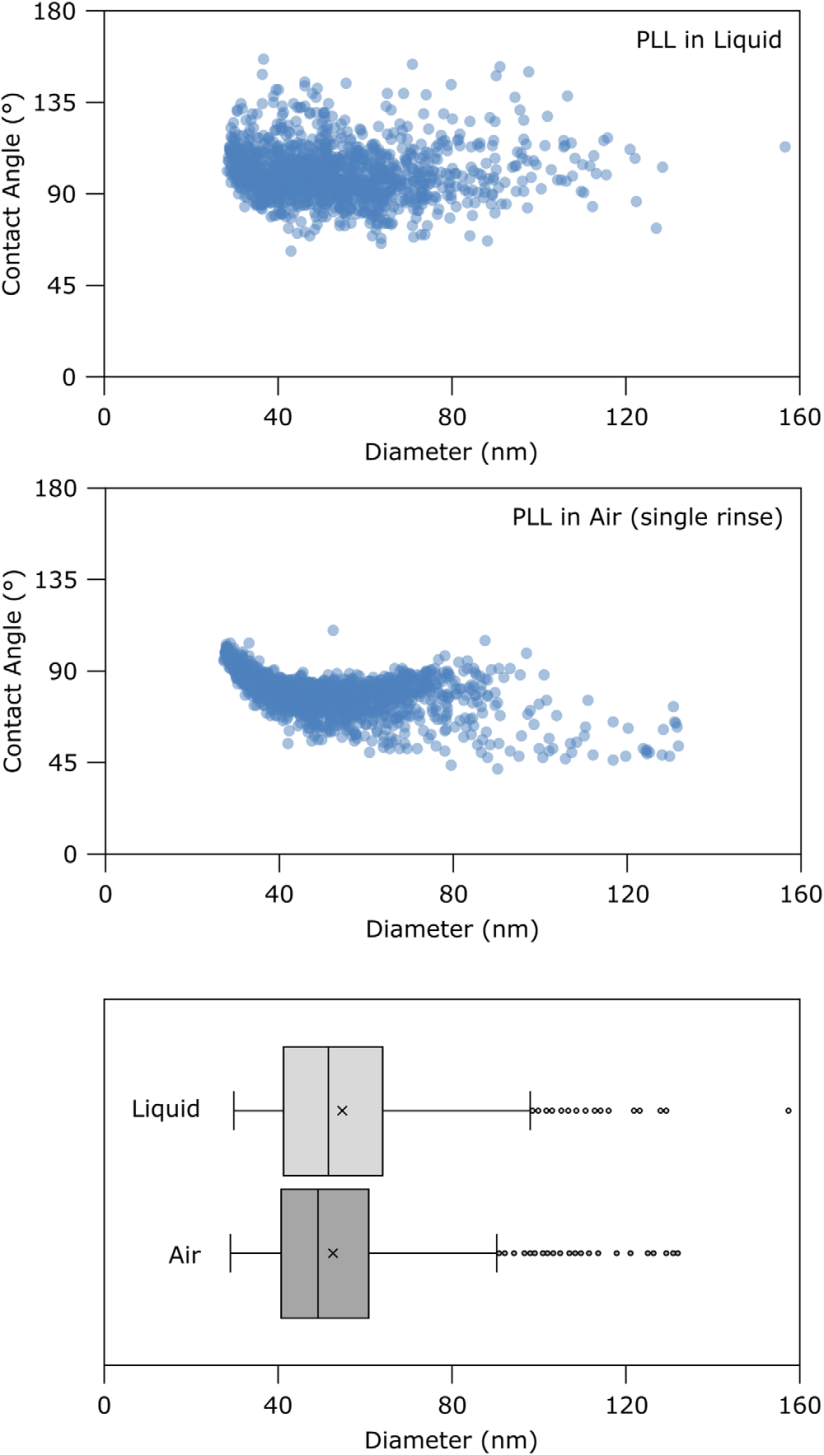
Single‐particle quantitative morphometry of individual particles found in aliquots from a single bovine milk EVs preparation deposited on 0.01 mg/mL PLL‐functionalised glass substrates and observed via AFM in liquid (top) or in air (middle). Each dot represents one particle. Contact angle and equivalent spherical diameter at constant surface were calculated as described elsewhere.^23^ Bottom: boxplot representation of the size distributions from the two scatterplots above, showing how they are almost completely coincident.

All of the results described in previous paragraphs refer to AFM imaging performed in liquid on PLL‐functionalised substrates. This choice is motivated by the fact that AFM imaging in air seems an intrinsically suboptimal choice for EVs, since it involves drying them on the substrate. While AFM does not need drying protocols as thorough as those used in EM, simply drying EV samples on a substrate often results in the co‐deposition of salts and/or amorphous materials (Figure 5). Moreover, samples are subjected to considerable shear stresses by the receding meniscus during drying, possibly resulting in an artificially low proportion of intact vesicles and an increased amount of non‐vesicular particles, or large surface density inhomogeneities across the substrate. Finally, the mechanical behaviour of a dried vesicle cannot be realistically assumed to correspond to that of its fully solvated state. Even so, despite the above considerations AFM imaging of EVs in air remains an extremely popular and widespread technique. In Figures 5 and 6, we show how the use of PLL‐functionalised substrates can drastically improve AFM images of EVs obtained in air up to the point of qualitatively resembling those obtained in liquid; however, we also show how several pieces of information regarding the sample are simply not available in these conditions.

AFM images of aliquotes from the same bovine milk EV^23^ batch differ markedly depending on deposition protocol and surface (Figure 5).^23^ The first image shows the result of drying a milk EVs aliquot on a freshly cleaved mica substrate. While globular particles that could correspond to putative intact EVs are visible, there is little else to infer from this image on a quantitative basis. The amount of salts and amorphous materials makes the background extremely rough, thereby preventing the univocal identification of a common baseline height for all particles, and consequently, any kind of quantitative morphometry. Since most EV samples show a log‐normal particle size distribution, and typically contain salts at concentrations orders of magnitude higher than those of bioparticles, the strategy of drastically diluting the sample to obtain isolated EVs on the bare substrate is not always feasible.

It is easy to see how AFM imaging in liquid after PLL capture performed on the same EV sample (Figure 5, second panel) results in a drastically cleaner micrograph, with all the associated advantages discussed in the previous sections.

However, a similar result can be obtained by following the same PLL‐based protocol EV deposition, but imaging the sample in air (see Section 2) after a controlled rinsing step followed by gentle drying with nitrogen (Figure 5, third panel). The resulting images are less ideal than those obtained in liquid, since their background contains more amorphous material; however, they also show numerous non‐adjoining globular particles and a clearly readable baseline height, which enables successive quantitative morphometry. Putative EVs show the same circular profile as that shown in Figure 1, which may allow them to be distinguished from amorphous material and non‐vesicular particles.

It is certainly possible to increase the number of rinsing steps (or their duration) to obtain images with a cleaner background; however, the overall quantity of adsorbed biomaterial *Q* strongly decreases with successive rinses (Figure 5, fourth panel). Due to this, it is considerably more difficult to reconstruct *Q* versus sample concentration plots such as the one shown in Figure 2B for experiments in liquid.

Quantitative AFM morphometry of aliquots from the same milk EV batch deposited on PLL‐functionalised substrates yields distinct results when imaging is performed in liquid versus air (Figure 6). As mentioned above, the mechanical behaviour of a non‐solvated vesicle is probably deposition‐dependent, and certainly different from those of EVs in solution. In these experiments for example, the average CA of dried EVs was shifted to markedly lower values than observed in liquid. Interestingly however, the EV diameter distribution is almost identical in both cases, suggesting that the morphometry algorithm we employ^23^, ^43^ also works on intact, dried EVs in exactly the same way. We have previously shown how AFM imaging performed in liquid can yield EV size distributions closely following those obtained via cryo‐EM^43^; the results summarised in Figure 6 suggest that the same result can be achieved with the technically simpler option of performing imaging in air.

## 4 CONCLUSION

We herein showed the impact exerted by some of the main experimental variables of a particle immobilisation protocol for subsequent EV morphometry previously employed in several papers.^24^, ^26^, ^33^, ^44^, ^46^, ^47^, ^48^ We showed how the parameter *Q* can be used as an estimator of relative sample concentrations across multiple depositions performed at constant PLL concentrations. We quantitatively substantiated the widespread usage of PLL 0.01 mg/mL as the most convenient. We also noticed how EV morphology as measured via CA appears to be independent from substrate charge density, a somewhat counterintuitive result that is nonetheless very useful in quantifying possible artefactual variations of CA due to deposition protocol variations as negligible. Finally, we have shown how PLL‐based EV surface capture can be used to significantly enhance the quality of AFM images in air, making quantitative morphometry, particularly for size distributions, achievable. While the mechanical fingerprint of individual EVs is theoretically lost in these conditions, CA distributions in air are still monomodal, suggesting that they might not be completely random; further studies are needed to substantiate this hypothesis.
